# Prediction of contaminant transport in fractured carbonate aquifer types: a case study of the Permian Magnesian Limestone Group (NE England, UK)

**DOI:** 10.1007/s11356-019-05525-z

**Published:** 2019-06-25

**Authors:** Giacomo Medici, Landis Jared West, Pippa Joanne Chapman, Steven Allan Banwart

**Affiliations:** 1 School of Earth and Environment, University of Leeds, Woodhouse Lane, Leeds, W Yorkshire LS2 9JT UK; 2 School of Geography, University of Leeds, Woodhouse Lane, Leeds, W Yorkshire LS2 9JT UK

**Keywords:** Carbonate aquifer, Fracture, Karst, Contaminant transport, Flowing porosity, Normal faults

## Abstract

Viruses and bacteria which are characterized by finite lives in the subsurface are rapidly transported via fractures and cavities in fractured and karst aquifers. Here, we demonstrate how the coupling of a robust outcrop characterization and hydrogeophysical borehole testing is essential for prediction of contaminant velocities and hence wellhead protection areas. To show this, we use the dolostones of the Permian Magnesian Limestone aquifer in NE England, where we incorporated such information in a groundwater flow and particle tracking model. Within this aquifer, flow in relatively narrow (mechanical aperture of ~ 10^−1^–1 mm) fractures is coupled with that in pipe cavities (~ 0.20-m diameter) following normal faults. Karstic cavities and narrow fractures are hydraulically very different. Thus, the solutional features are represented within the model by a pipe network (which accounts for turbulence) embedded within an equivalent porous medium representing Darcian flowing fractures. Incorporation of fault conduits in a groundwater model shows that they strongly influence particle tracking results. Despite this, away from faulted areas, the effective flow porosity of the equivalent porous medium remains a crucial parameter. Here, we recommend as most appropriate a relatively low value of effective porosity (of 2.8 × 10^−4^) based on borehole hydrogeophysical testing. This contrasts with earlier studies using particle tracking analyses on analogous carbonate aquifers, which used much higher values of effective porosity, typically ~ 10^2^ times higher than our value, resulting in highly non-conservative estimates of aquifer vulnerability. Low values of effective flow porosities yield modelled flow velocities ranging from ~ 100 up to ~ 500 m/day in un-faulted areas. However, the high fracturing density and presence of karstic cavities yield modelled flow velocities up to ~ 9000 m/day in fault zones. The combination of such flow velocities along particle traces results in 400-day particle traces up to 8-km length, implying the need for large well protection areas and high aquifer vulnerability to slowly degrading contaminants.

**Electronic supplementary material:**

The online version of this article (10.1007/s11356-019-05525-z) contains supplementary material, which is available to authorized users.

## Introduction

Fractured carbonate aquifers, which are subjected to different degrees of karstification, underlie a land area covering ~ 15% of the earth’s surface and supply ~ 25% of the world’s population with drinking water (Hartmann et al. 2014). A range of pollutants can reach the saturated part of these aquifers in regions which are devolved to industry and agriculture, including nitrate, sulphate, chloride, toxic organic compounds released by mineral fertilizers and pesticides, and pathogens (Bales et al. 1989; Göppert and Goldscheider 2008; Mondal and Sleep 2013; Petitta et al. 2009, 2018; Reh et al. 2015; Ducci et al. 2017; Yang et al. 2017; Liu et al. 2018; Marijić et al. 2018). Animal excreta applied directly to the land from grazing animals or applied to land as farm yard manure or slurry represent a key source of nitrate, viruses, and bacteria (Conboy and Goss 2000; Wakida and Lerner 2005; Rivett et al. 2008).

Viruses, bacteria, and many chemical contaminants such as pesticides are characterized by specific subsurface survival times; exposure of receptors can occur where the time taken to reach supply wells or springs does not exceed these survival times and therefore depends on their velocity of transport in the subsurface (Pekdeger and Matthess 1983). Unfavourably, fast transport of these pollutants occurs in correspondence of bedding plane discontinuities, joints, and fault-related fractures rather than via porous matrix in most lithified sedimentary rocks (Hitchmough et al. 2007; Lo et al. 2014; Kocabas and Bulbul 2015; Medici et al. 2016, 2018; Jones et al. 2017). As a consequence, accurate predictions of travel time of viruses and bacteria through rock discontinuities via groundwater flow and contaminant transport models are needed in order to define groundwater source protection areas around springs and abstraction boreholes (Taylor et al. 2004; Riva et al. 2005; Bagherzadeh et al. 2018). The delineation of source protection areas and/or well capture zones allows regulators to restrict land use and activities such as manure spreading to avoid impacts on sources.

In this paper, we show how the coupling of robust characterization of rock discontinuities in an outcrop and hydrogeophysical borehole testing makes a large difference in the prediction of contaminant transport velocities and therefore the vulnerability of borehole abstraction points to pollution. We also provide guidelines on how to better predict transport in dolomitic limestone environments using both appropriate values of flowing porosity in un-faulted areas and a flow regime in correspondence of normal faults. Although these are key aspects of management of carbonate aquifers, geo-modellers in industry, research institutes, and universities typically treat fault hydraulics and flowing porosity in a non-rigorous way. Indeed, prediction of contaminant transport using codes such as MODPATH requires specification of the effective flowing porosity to define source protection areas (Pollock 2016). Values assigned to this parameter range from ~ 10^−3^ to 10^−1^ in fractured carbonate aquifers with a typical value of 5.0 × 10^−2^ which is assigned by modellers without evidence from hydrogeophysical tests (Neymeyer et al. 2007; Bredehoeft and King 2010; Worthington et al. 2012; Yager et al. 2013; Zuffianò et al. 2016; Gárfias et al. 2018). In contrast, the combination of an acoustic televiewer and fluid logging with hydraulic conductivity from falling or rising head tests shows much lower values (~ 10^−4^) of effective porosity in fractured rocks (Quinn et al. 2011; Ren et al. 2018). Values of effective porosity in the same order of magnitude have been found by Maldaner et al. (2018) in a fractured dolostone combining an acoustic televiewer and well dilution tests with falling and rising head and pumping test data. Similar results were obtained for our case study aquifer by Medici et al. (2019a) in the fractured dolomitic limestones of the UK Magnesian Limestone aquifer which is the subject of this paper. In this paper, we test the sensitivity of contaminant transport to flowing porosity values assumed for this aquifer.

Faulted areas in limestone environments typically show high values of transmissivities, and springs often emerge from associated systems of conduits or caves (Allen et al. 1997; Amoruso et al. 2013; Maurice et al. 2016; Bauer et al. 2016). As a consequence, several authors highlight the need to model turbulent flow in correspondence of faults in karst environments (Bauer et al. 2003; Hill et al. 2010; Gallegos et al. 2013; Saller et al. 2013). Faults in high mechanically (UCS_nat_ > 45 MPa) resistant carbonate rocks are characterized by either non-intensively karstified fractures or karstic cavities (Billi et al. 2007; Lott 2013; Bauer et al. 2016). Flow can be Darcian in correspondence of relatively closed fault-related fractures (Berkowitz et al. 1988; Berkowitz 2002). However, cavities in correspondence of normal faults can approximate pipes in which turbulence arises (Worthington and Ford 2009; Hill et al. 2010; Gallegos et al. 2013; Saller et al. 2013). To account for this, in this paper, we model both Darcian and non-Darcian (turbulent) flow in fault zones using the available MODFLOW-2005 numerical codes.

**Fig. 1:**
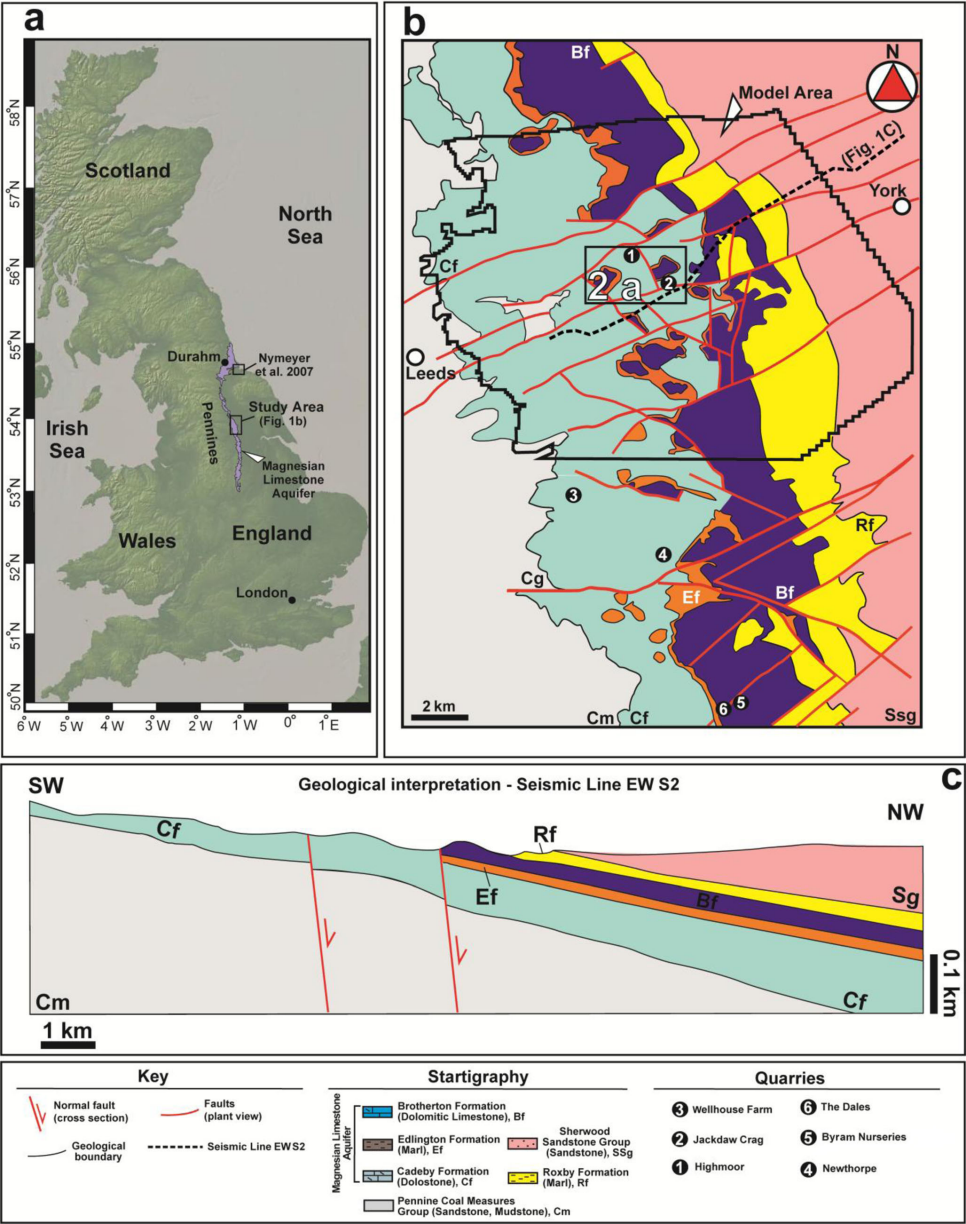
Study area. **a** Map describing the Magnesian Limestone aquifer in Great Britain and the location of the study area (basemap from GeoMapApp) and that of a previous study by Neymeyer et al. (2007). **b** Geological map with the location of the numerical groundwater flow model area (thick black line) and the University of Leeds farm site for hydrogeophysical characterization (boxed area, see the enlarged view in Fig. 2) and discontinuity surveys (numbers) and the location of the cross section shown in **c** (dashed line). **c** The geological cross section EW-S2 based on a seismic line (Cooper and Lawley 2007)

Scientific literature on the physical hydrogeology of the Magnesian Limestone aquifer in Great Britain has focused on core plug and pumping test analyses (Aldrick 1978; Cairney 1972; Allen et al. 1997). More recently, Medici et al. (2019a) combined optical and televiewer logging, monitoring of the water table, slug tests, and temperature and electrical conductivity fluid logs to compute groundwater flow velocities in the area of this study in Yorkshire, UK (Fig. 1a, b). Time series data show minimal seasonal variation of groundwater flow velocities and direction (Medici et al. 2019a). As a consequence, steady-state flow models can adequately represent the flow velocity field. Previous published geochemical data are limited to analyses of major anions and cations in the Magnesian Limestone aquifer in the County Durham area, UK (Crabtree and Trudgill 1984; Younger 1995; Mayes et al. 2005). In this paper, baseline hydrochemical analyses that provide data on pCO_2_ and saturation indexes of calcite, dolomite, and aragonite for the area of this study (Fig. 1a, b) are also presented. Our purpose is to provide useful information on drivers of karst development in correspondence of rock discontinuities (bedding planes, joints, and faults) which represented the focus of the flow modelling in this research.

The overall aim of this paper is to improve the prediction of contaminant transport in fractured carbonate aquifers showing karst development, based on the incorporation of outcrop and borehole hydrogeophysical data within groundwater flow and particle tracking models. The specific objectives of the presented research are (i) to establish a link between hydrochemistry and karstification in the Magnesian Limestone aquifer, (ii) to achieve a reliable groundwater flow model that accounts for outcrop and hydrogeophysical constraints on the key flow pathways, (iii) to identify the most appropriate parameters and approaches for particle tracking both away from and around faults, and (iv) to identify the key structural and hydraulic factors that influence contaminant transport.

## Study area

### Geological background

**Fig. 2:**
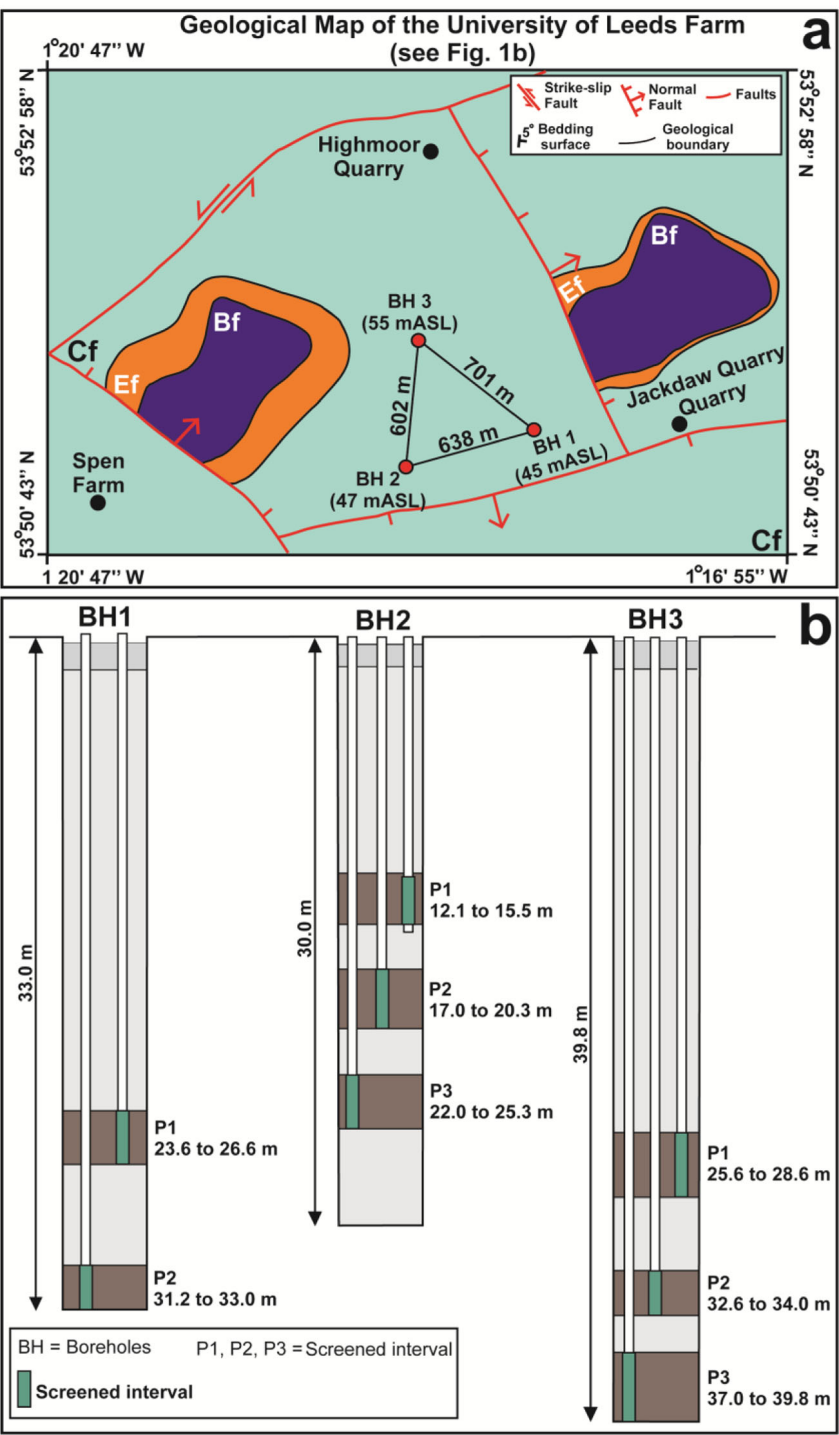
University of Leeds experimental farm site showing the location and elevation of the three drilled boreholes where monitoring/hydrogeophysical characterization was undertaken in 2017/18. **a** Geological map and location of boreholes (see Fig. 1 for legend). **b** Geometry of BH1, BH2, and BH3 piezometer installations

The study area (see Fig. 1a–c) is located in Yorkshire (NE England, UK) between the cities of Leeds and York. This area includes both the site of detailed hydrogeophysical characterization undertaken by Medici et al. (2019a) and that of the flow model developed in this paper (Figs. 1b and 2a). The Magnesian Limestone Group is formed by dolomitic limestone, dolostone, and evaporite rocks derived from sedimentation in the marginal areas of the Permian Zechstein Basin (Harwood 1986; Smith et al. 1986). In Yorkshire, the Magnesian Limestone aquifer was deposited during the Lower Permian under subtropical and tropical conditions. The Magnesian Limestone Group is formally part of the Zechstein Group in NE England (Harwood 1986). Here, this group is characterized by three geological formations: the Cadeby, Edlington, and Brotherton formations (Fig. 1b; Smith et al. 1986).

The Cadeby and Brotherton formations are carbonate units; they are separated by the anhydrite and gypsum strata of the Edlington Formation (Fig. 1b). The basal part of the Cadeby Formation is characterized by a 5-m-thick marl interval which separates the geological formation from the marly sandstones and clays of the Pennine Coal Measures Group (Fig. 1b, c). The Brotherton Formation passes upwards into the Roxby Formation which represents an aquiclude formed by anhydrite, gypsum, and mudstone (Cooper 1988).

The Cadeby Formation is dominated by wackstones, packstones, and grainstones showing ooids, corals, and bivalves in a thin section (Kaldi 1986). This formation was deposited during the Lower Permian (272–251 Ma) in a shallow marine environment. Then, secondary dolomitization occurred during the early Permian and middle Triassic time (Harwood 1986; Kaldi 1986; Peryt and Scholle 1996). Dolomitization fluids likely originated from the gypsum, anhydrite, and halite of the overlying Edlington Formation (Harwood and Coleman 1983; Harwood 1986). The Brotherton Formation above represents the uppermost unit of the Magnesian Limestone aquifer in Great Britain and is formed by thin dolomitic limestone beds with ooids, peloids, photosynthetic algae, and micro- and macro-foraminifera. The wackstones, packstones, and grainstones of the Brotherton Formation were also deposited in a shallow water environment, but they differ from those of the Cadeby Formation due to less intense dolomitization (Harwood 1986; Peryt and Scholle 1996). Indeed, the Cadeby Formation (54% CaCO_3_, 46% MgCO_3_) is less abundant in calcite with respect to the Brotherton Formation (54% CaCO_3_, 46% MgCO_3_) in NE England (Kaldi 1986; Lott and Cooper 2005).

In Yorkshire, in the area of study (Fig. 1b, c), the tectonic features which deform the UK Magnesian Limestone aquifer are extensional faults and non-stratabound joints (sensu Odling et al. 1999). Quarry observations and seismic reflection surveys carried out in the Leeds-York area show how extensional faults of the Mesozoic age are mainly oriented ENE-WSW (Fig. 1b; Walsh and Watterson 1988; Cooper and Lawley 2007). The absence of major normal faults oriented parallel to the Pennine structures in NE England is due to lack or limited extension related to the Permo-Mesozoic opening of the Atlantic Ocean (Hallam 1971; Burley 1984; Medici et al. 2015, 2019b). Lack of significant effects of the Cenozoic Alpine orogenesis results in the gentle dip (< 5° towards E) of the Permo-Mesozoic deposits in the Yorkshire area (Fig. 1b, c; Bottrell et al. 2006; West and Truss 2006; West and Odling 2007; Keim et al. 2012: Medici et al. 2019a). The Magnesian Limestone aquifer varies in thickness from 70 to 110 m towards the east (see Fig. 1c). This is due to the shelf-edge geometry of the Permo-Triassic basin in the study area which overlaps on the Carboniferous strata further west (Cooper and Lawley 2007; Medici et al. 2015).

Field observations at quarries in the Cadeby and Brotherton formations show how normal faults are characterized by zones of deformation up to ~ 30 m wide in sections parallel to the dip (Aldrick 1978; Allen et al. 1997). However, boreholes drilled in non-faulted sections show high angle joints (dip 50–80°) which cross-cut the bedding parallel fractures (Medici et al. 2019a). Such high angle joints have been previously studied in the Magnesian Limestone aquifer in County Durham (Kortas and Younger 2013) as well as in other aquifers of the Permo-Mesozoic age across Great Britain (Allen et al. 1997, 1998; Hitchmough et al. 2007; Medici et al. 2016, 2018). These fractures arise from the Cenozoic uplift of NW Europe and are related to unloading due to removal of overlying beds (Odling et al. 1999; Gillespie et al. 2001; Kortas and Younger 2013). Other discontinuities present in the Magnesian Limestone are subhorizontal bedding plane fractures and stylolites. Bedding plane fractures in this aquifer originate from movements associated with the Cenozoic uplift (Kortas and Younger 2013). Stylolites are solution features that formed during burial diagenesis under the effect of the lithostatic pressure (Railsback, 1983; Tondi et al. 2006).

### Hydrogeological setting

The Magnesian Limestone aquifer represents the fifth most important aquifer for volumes of water withdrawn in Great Britain (Younger 1995; Edmunds et al. 2003; Rivett et al. 2007; Abesser and Lewis 2015). Contamination in this dolomitic aquifer is related to the application of fertilizers to agricultural fields. Indeed, nitrate concentrations steadily increased after the Second World War from an average of 0.30 mg L^−1^ in 1945 to 80 mg L^−1^ in 2005 in the study area (Aldrick 1978; Walker 2006). The Magnesian Limestone aquifer is unconfined in the western part of the area due to the absence or presence of only ~ 1-m-thick superficial cover consisting of sands and clays of the Quaternary age (Fig. 1b; Gaunt et al. 1970; Allen et al. 1997). The aquifer becomes confined in the eastern part of the groundwater flow model area (Fig. 1b), i.e., resulting from gentle dip towards the east (see Fig. 1c). The Cadeby and Brotherton formations represent dolomitic, relatively permeable units, which are separated by the marls and evaporates of the Edlington Formation. The latter formation is considered a leaky aquitard; i.e., dissolution of anhydrite and gypsum beds only gives very limited permeability (Farrant and Cooper 2008). Boreholes with piezometers installed in both the Cadeby and Brotherton formations show head differences ranging from 0.25 up to 2.00 m in some areas of NE England (Aldrick 1978; Allen et al. 1997). However, some hydraulic connectivity between the Cadeby and Brotherton formations arises from normal faulting (Farrant and Cooper 2008). The Magnesian Limestone aquifer is highly mechanically resistant (UCS of 48–75 MPa), and thus it appears to be heavily fractured at quarry sites (Lott and Richardson 1997; Cooper and Lawley 2007; Lott 2013).

Groundwater flow is directed towards the east driven by topography which is characterized by the topographic high of the Pennines to the west (Fig. 1a). The Cadeby Formation interquartile interval ranges for porosity and core plug hydraulic are 8.5 to 18.7% and 2.9 × 10^−4^ to 0.9 × 10^−3^, respectively. These interquartile intervals largely overlap those of the Brotherton Formation, which are 9.9–19.0% and 4.0 × 10^−4^–1.5 × 10^−3^ m/day for porosity and core plug scale hydraulic conductivity, respectively (Allen et al. 1997).

Slug tests in the Cadeby Formation of the UK Magnesian Limestone show hydraulic conductivities ranging from 0.07 up to 2.89 m/day. Notably, higher values of hydraulic conductivities (*K* = 0.83–2.89 m/day) from these tests characterize the first ~ 15 m below the water table (Medici et al. 2019a). Single-borehole pumping test values from the Magnesian Limestone aquifer of Great Britain range from 6 to 300 m^2^/day with a median of 25 m^2^/day in un-faulted areas (Cairney 1972; Allen et al. 1997).

Assuming a ~ 15-m effective thickness for the Cadeby Formation in the model area (Fig. 1b), hydraulic conductivity ranges from 0.8 to 10 m/day with median values of 1.3 m/day in un-faulted areas; the hydraulic conductivity of the Cadeby Formation (*K*_median_ = 1.2 m/day, *n* = 16) is lower with respect to that of the Brotherton Formation (*K*_median_ = 2.7 m/day, *n* = 15) (Medici et al. 2019a). This has been considered a consequence of the higher dolomitization which characterizes the Cadeby Formation; this reduces the rate of mineral dissolution along rock discontinuities. By contrast, the dolomitic limestone of the Brotherton Formation is more abundant in calcite; thus, dissolution and development of permeable flow pathways are favoured (Cooper and Lawley 2007; Farrant and Cooper 2008).

The highest transmissivity (*T*_median_ = 2000 m^2^/day; *n* = 7) values in the Magnesian Limestone in both the Cadeby and Brotherton formations are related to extensional faults (Aldrick 1978; Allen et al. 1997; Cooper and Lawley 2007; Neymeyer et al. 2007). Springs emerge in correspondence of faults at the study site between the cities of Leeds and York (Aldrick 1978). Groundwater flow is largely dominated by fractures. This is shown by a four-order magnitude difference between hydraulic conductivity from well and core plug tests (*K*_well-test_/*K*_core-plug_ ~ 10^4^; Allen et al. 1997). Groundwater in the Cadeby Formation in the northern area of Durham (Fig. 1a) is highly alkaline (380–450 mg L^−1^), due to chloride and sulphate dissolution from the anhydrite and halite of the Edlington Formation above (Mayes et al. 2005).

## Material and methods

### Scanlines

Horizontal and vertical scanline surveys have been carried out at six quarry outcrops around the Leeds-York area in Yorkshire (see Fig. 1a, b) to characterize the fracture network of the Magnesian Limestone Group in faulted and un-faulted sections. The selected method (Hitchmough et al. 2007) consists in the recording of 7 parameters in an outcrop: strike orientation, dip inclination, fracture spacing, percentage of infilled fractures, fracture trace persistence, and mechanical aperture. Overall, 401 discontinuities were recorded and divided into 6 groups (D1–6): bedding planes (D1, *n* = 114), stylolites (D2, *n* = 25), south (D3, *n* = 67) and north (D4, *n* = 90) dipping subvertical joints, and south (D5, *n* = 60) and north (D6, *n* = 45) dipping fault-related fractures. Rock discontinuities were plotted on stereonets, and mean vector statistics of each group computed with the Stereonet software package (Allimendinger et al. 2012).

### Physiochemical analyses

Physical and geochemical parameters of groundwater have been assessed in three boreholes (BH1, BH2, and BH3) drilled at the University of Leeds farm (Fig. 1b, c). Here, groundwater sampling for physiochemical analyses has occurred on six occasions during 2018.

Temperature, conductivity, pH, and alkalinity were recorded in the field on samples collected from each screened interval (see Fig. 2a, b) of the three boreholes using a discrete interval bailer (425 LDPE Solinst). Temperature and pH were measured using 6PFCE Ultrameter II (Myron L Company); electrical conductivity and alkalinity (i.e., bicarbonate HCO_3_^−^) were measured using a 9033 Multi-Range Electrical Conductivity Meter (Hanna Instruments) and Digital Titrator Model 16900′ (Hach Company), respectively. Calibration of probes was carried out at the beginning of each day of field work, and maintenance of calibration was checked prior to each measurement. Groundwater samples were filtered through a 0.25-μm pore membrane into a 50-mL bottle in the field using a syringe; a few drops of 10% nitric acid were added to the 50-mL bottles for preservation of cations. Samples were preserved at 4 °C in the fridge prior to laboratory analyses (Piper 1953). Major anions (Cl^−^, SO_4_^2−^, NO_3_^−^) and cations (Na^+^, Mg^2+^, K^+^, Ca^2+^, Mn^2+^, Fe^2+^, Al^3+^) were determined in the laboratory using an iCAP™ 7600 ICP-OES and ICS-3000 Ion Chromatographer (both Thermo Scientific Ltd.), respectively. Charge balance errors were calculated and range from 0.1 up to 3.8%, which suggests good-quality data. Dissolved organic carbon (DOC) was calculated from the difference between total dissolved carbon (DC) and dissolved inorganic carbon (DIC), which were both measured by a Multi N/C 2100 Analyser (Analytik Jena).

PHREEQC 3.4.0 Interactive (Parkhurst and Appelo 1999) was used to calculate the partial pressure of carbon dioxide (pCO_2_) and saturation indexes (SI) for calcite, aragonite, and dolomite.

### Groundwater flow model

A steady-state groundwater flow model of the Magnesian Limestone aquifer was developed for the Leeds-York area of NE England (Fig. 1b) by the Environment Agency using MODFLOW-2005 (Environment Agency 2009). In this study, this model was modified to take account of the hydrogeophysical data on the effective aquifer thickness of the Cadeby Formation, which is much less than its full thickness (Medici et al. 2019a), and karst development in the UK Magnesian Limestone associated with faults.

The Environment Agency (2009) steady-state flow model was calibrated using hydraulic conductivity and river bed conductance. This model simulates steady-state flow conditions from 1 February 2004 to 31 December 2008, using hydraulic heads measured by the Environment Agency from 47 observation boreholes within the Cadeby Formation (Fig. 8c).

The 3D groundwater flow model is characterized by three layers which represented the Cadeby (lowest, layer 1), Edlington (layer 2), and Brotherton (uppermost, layer 3) formations of the UK Magnesian Limestone, respectively.

External boundary conditions are the same in layers 1 and 2 of the Environment Agency (2009) steady-state flow model for the northern, southern, and western edges. The northern edge is a no-flow boundary, as the drainage network indicates a groundwater divide. The southern boundary is treated as a general head boundary due to the continuation of the Magnesian Limestone aquifers beyond the modelled domain here and the absence of a groundwater divide. The western edge of the steady-state groundwater flow model is also considered a general head boundary to account for cross-boundary flow with the underlying Carboniferous strata (see Fig. 1b). The eastern edge is defined as a no-flow boundary for the Cadeby (layer 1) and Edlington (layer 2) formations which lie within the deeply confined part of the aquifer beneath the Roxby Formation (Upper Permian Marl, see Fig. 1b), so flow is likely to be negligible. However, the uppermost Brotherton Formation (layer 3) in the eastern edge is represented as a general head boundary to represent flow to/from the Sherwood Sandstone Group through the Roxby Formation (see the geological structure in Fig. 1b, c).

Rainfall recharge is applied to the uppermost layer in the model at any given location and was calculated from four sources: precipitation and evapotranspiration data, a map of Quaternary deposits, and soil type (Environment Agency 2009). Note that hydraulic head data and boundary conditions are unchanged in the new version of the groundwater flow model described below.

New analysis of rock discontinuities coupled with previous geomorphological observations (Murphy 2000; Cooper and Lawley 2007) was also used to modify the Environment Agency (2009) model. The Conduit Flow Process Mode 1 (CFPM-1) pipe flow framework developed by Hill et al. (2010) for MODFLOW-2005 was inserted, due to the presence of faults, to model turbulence in conduits observed in outcropping fault zones. A CFPM-1 pipe network composed of 1317 nodes was created following the recommendations of the United States Geological Survey (USGS) (Gallegos et al. 2013; Saller et al. 2013). Rates of laminar (*Q*_l_) and turbulent (*Q*_*t*_) flow for the pipes are described by Eqs. (1) and (2), respectively (Hill et al. 2010; Saller et al. 2013):

1 $$ {Q}_1=-\frac{d^4\pi g\Delta h}{128v\Delta l\tau} $$

2 $$ {Q}_t=-\frac{\Delta h\ }{\mid \Delta h\mid}\sqrt{\frac{\mid \Delta h\mid g{d}^5{\pi}^2}{2\Delta l\tau}\ }\log \frac{2.5v\Delta l}{\sqrt[4]{\frac{2\mid \varDelta h\mid g{d}^3}{\varDelta l\tau}}}+\frac{r}{3.71d} $$

In the previous equations, *d* (L) is the pipe diameter, *τ* (−) is the pipe tortuosity, *r* (L) is the average micro-topography (or asperity height) of the pipe walls, *ν* (L^2^ T^−1^) is the kinematic viscosity, *g* (LT^−2^) is the gravitational acceleration constant, Δ*l* (L) is the pipe length, and Δ*h* (L) is the hydraulic head loss along the pipe. Δ*l* is 100 m equal to the length of a cell. The diameter (*d*) of the pipes in the network has been assigned by model calibration. Pipe tortuosity has been assigned a value of 1.3 based on quarry outcrop observations (Boddy 2018; Walker 2006). Kinematic viscosity of water was assigned a value of 1.31 × 10^−6^ m^2^ s^−1^, based on the annual average water temperature of 10 °C in boreholes with seasonal variation < 1.5 °C (Environment Agency 2009). The average micro-topography (*r* = 1.8 × 10^−3^ m) of the pipe walls is known from previous measurements on rock samples from quarries in the area (Boddy 2018).

### Particle tracking analysis

Abstraction well capture zone analysis was carried out using the MODPATH particle tracking code (Pollock 2016). Backward particle tracking analysis is run for 100 particles from the 4 abstraction boreholes. The MODPATH code uses the cell-by-cell flow terms that are created by the MODFLOW-2005 simulation to determine backward particle movement directions and rates, along with the effective flow porosity.

The MODPATH code has been applied to the new groundwater flow model which was produced implementing the pre-existing model (Environment Agency 2009). Effective flow porosity value was set to either 2.8 × 10^−4^ or 5.0 × 10^−2^ which are values for UK Magnesian Limestone. The value of 2.8 × 10^−4^ was determined from well acoustic and fluid logging combined with slug tests by Medici et al. (2019a) at the University of Leeds farm site within the modelled area (Figs. 1b and 2a). The much higher value of 5.0 × 10^−2^ was used in particle tracing modelling of the County Durham Magnesian Limestone by Neymeyer et al. (2007). This is a standard value from the literature for such aquifer types (Bredehoeft and King 2010; Zuffianò et al. 2016; Gárfias et al. 2018).

For each effective flow porosity, particle traces have been found for two different travel times of 50 and 400 days. These two travel time values are based on short- and long-lived pathogens in groundwater for definition of wellhead protection areas (Taylor et al. 2004). Note that particle tracking analysis exclusively involves the Cadeby Formation due to the position of the abstraction wells.

## New characterization of the Magnesian Limestone aquifer

### Fracturing network

**Fig. 3:**
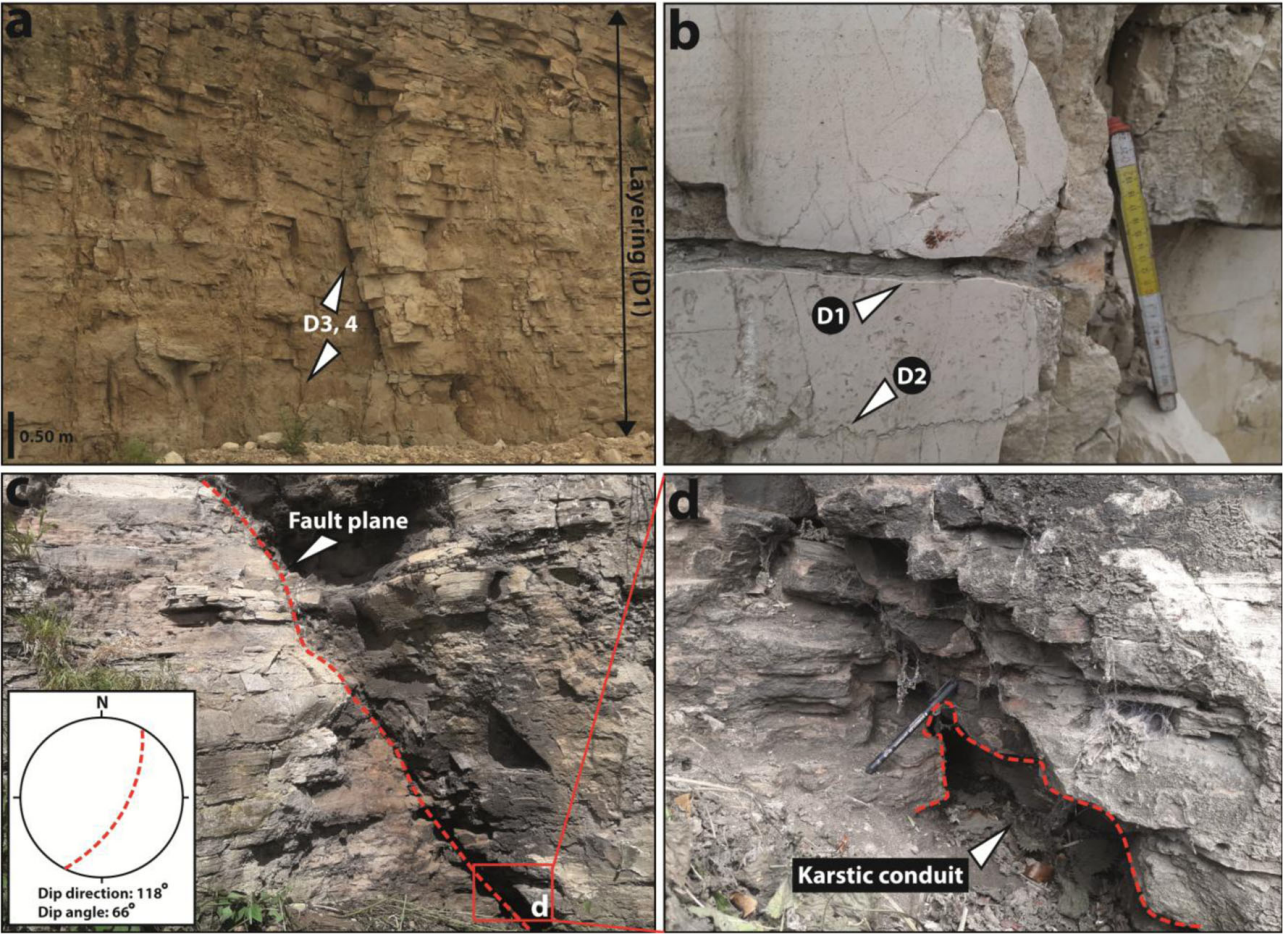
Outcropping Magnesian Limestone Group (see Fig. 1b for the location of quarries). **a** Cadeby Formation at Jackdaw Crag Quarry. **b** Detail of bedding fracture (D1) and detail of stylolite (D2) in the Cadeby Formation at Highmoor Quarry. **c** Extensional fault in the Brotherton Formation outcropping in ‘the Dales Quarry’. **d** Karstic conduit in the fault zone at ‘the Dales Quarry’, Brotherton Formation

**Fig. 4:**
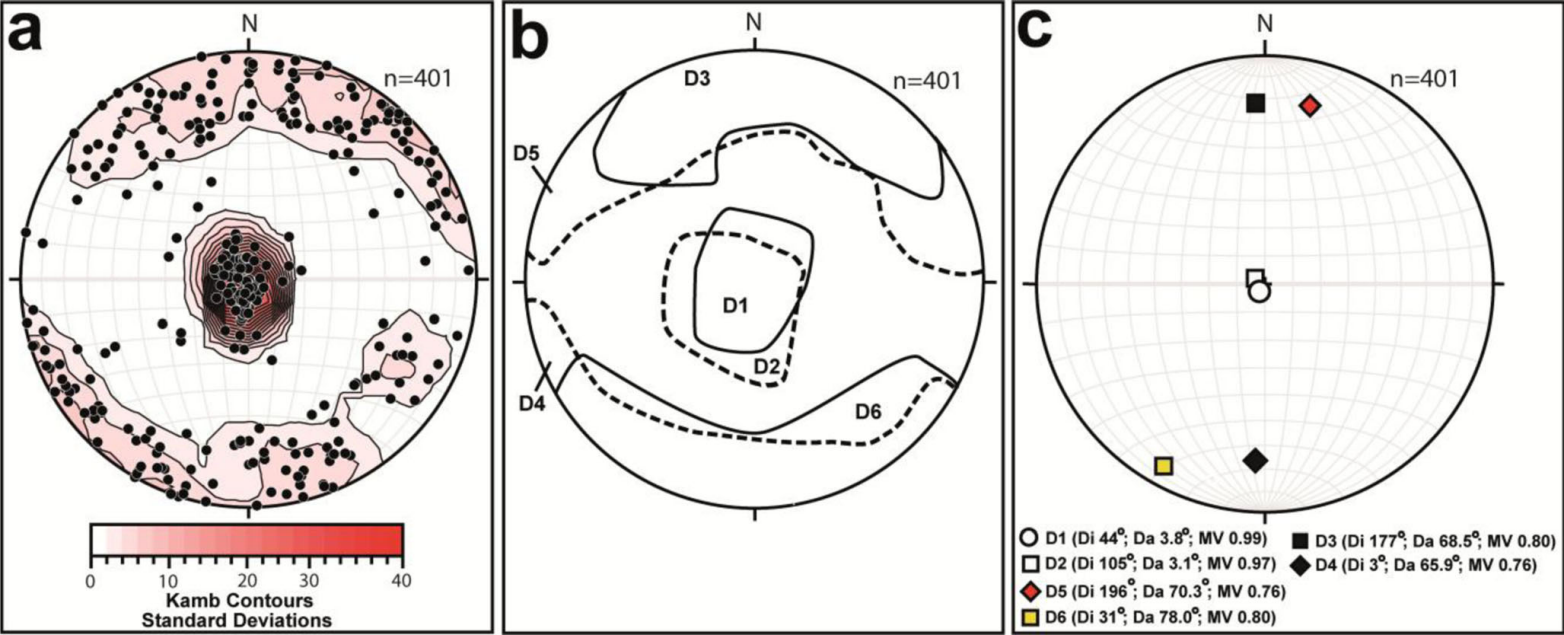
Stereoplots of pole to planes (upper hemisphere, equal area) from 14 scanline surveys carried out in 6 different quarries in the Leeds-York area. **a** Contours. **b** Grouping of discontinuities (D1–D6). **c** Mean vectors of discontinuities (D1–D6)

Visual observations of karst features and discontinuity surveys (scanlines along both horizontal and vertical directions) have been performed in quarries (see Fig. 1b for locations, Figs. 3a–d for photos, and Table 1 and Figs. 4 and S1 for scanline results) in the Cadeby (Jackdaw, Highmoor, Newthorpe, Wellhouse Farm, and Byram Nurseries quarries) and the Brotherton (Byram Nurseries and the Dales quarries) formations of the Magnesian Limestone Group.

**Table 1:** Details of scanline data acquired in the Magnesian Limestone Group in the Leeds-York area (see Fig. 1b for the location). The initial uppercase letter of the scanline code represents the quarry site initial, lower case h and v represent horizontal and vertical scanlines, and numbers 1 and 2 indicate horizontal surveys in orthogonal directions

Field site	Population (*n*)	Scanline code	Face orientation	Scanline length (m)	Average spacing (m)	Standard deviation, *σ* (m)
Jackdaw Crag Quarry	31	J, h	35–215°	9.0	0.27	0.08
Highmoor Quarry	52	H h1	125–305°	7.0	0.13	0.01
	8	H h2	20–200°	1.0	0.12	0.06
	46	H v	110–290°	4.0	0.09	0.09
Newthorpe Quarry	28	N h	120–210°	4.0	0.20	0.01
	12	N v	125–215°	1.8	0.15	0.01
Wellhouse Farm Quarry	12	W h1	2–182°	2.0	0.15	0.06
	14	W h2	50–230°	2.0	0.14	0.12
	9	W v	90–270°	2.5	0.26	0.22
The Dales	26	D h1	102–282°	1.5	0.05	0.06
	12	D h2	10–190°	1.4	0.09	0.07
	24	D v	115–295°	1.3	0.04	0.03
Byram Nurseries	27	B h1	12–192°	4.0	0.15	0.08
	80	B h2	20–200°	7.0	0.14	0.08
	20	B v	20–120°	1.0	0.05	0.04

The fracturing network in un-faulted sections has been studied at Jackdaw Crag, Highmoor, Newthorpe, Wellhouse Farm, and Byram Nurseries quarries. Normal faults are not mapped (Fig. 1b) in Highmoor, Newthorpe, and Wellhouse Farm quarries, and they have not been detected in our surveys there. Although normal faults outcrop at Jackdaw Quarry, such structures have been avoided, i.e., poor exposure of these tectonic structures. Scanline surveys have been performed both on un-faulted (Bh1, Bv) and faulted (Bh2) rock faces at the Byram Nurseries quarry (see Table 1). Scanline surveys (Dh1, Dh2, Dv) were performed in correspondence of a normal fault at the Dales quarry (see Fig. 1b and Table 1).

Discontinuities were subdivided into six different sets (Fig. 4b). Bedding planes (D1) and stylolites (D2) which are subhorizontal (< 5°) are related to the sedimentary structure of the Magnesian Limestone Group. Open bedding plane (D1) discontinuities represent either bedding planes enlarged by groundwater dissolution or mechanically reactivated fractures which are due to the Cenozoic uplift of Great Britain (Kortas and Younger 2013).

Stylolites (D2) are pressure solution structures which are characterized by more irregular shape with respect to the bedding plane (D1) discontinuities (see Fig. 3b). Stylolite (Fisher mean vector = 105° − 3.1°) and bedding plane (Fisher mean = 44° − 3.8°) dip directions are consistent with the regional dip towards the northeast or east (Figs. 1c, 4a–c, and S1).

South (D3) and north (D4) dipping high angle (55 to 90°) joints are interpreted as related to the Cenozoic uplift of NW Europe in the Magnesian Limestone Group (Kortas and Younger 2013). However, fractures dipping towards south (D5) and north (D6) are related to the extensional faults outcropping at the Dales and the Byram Nurseries quarries (Fig. 1b). Plotting of fault-related (D3, D4) and non-fault-related (D5, D6) discontinuities shows how both are E-W striking (Figs. 4 and S1).

The average vertical spacing of bedding plane (D1) discontinuities is 0.20 m (Fig. 3a); the average persistence of non-fault-related subvertical joints (D3, D4) is 0.66 m (Table 2). Subvertical joint persistence exceeds vertical spacing of bedding fractures. Thus, bedding plane fractures (D1) and subvertical joints (D3, D4) collectively form a non-stratabound fracturing network. Fault-related fractures (D5, D6) are similar to the non-stratabound joints (D3, D4) in terms of persistence (see Table 2). However, the fault zones outcropping at the Dales and Byram Nurseries quarries are characterized by a higher density of such joints with respect to un-faulted sections in the Magnesian Limestone Group (see Table 1). The average spacing of fractures in a fault zone is 0.04 and 0.11 m along the vertical and horizontal directions, respectively. This contrasts with non-faulted sections which are characterized by higher average vertical (0.05 m) and horizontal (0.17 m) spacing (Table 1). Fault-related fractures (D5, D6) also differ from the non-stratabound fracturing in that they have much higher fracture aperture (Table 2). The average aperture (*a*) is 1.6 mm in the fault zones of the Dales and Byram Nurseries quarries. Here, some apertures in correspondence of faults range from 0.10 up 0.6 m (see cavities in Fig. 3c, d). Cavities show a range of cross sections in an outcrop from circular and tabular to polygonal with smoothed angles. Although such cavities are likely enlarged by groundwater dissolution, note that 58% of discontinuities in faulted zones nevertheless show apertures < 1 mm. Away from faults, the average aperture is 0.7, 0.6, and 0.3 mm for non-stratabound joints (D3, D4) and bedding planes (D1), respectively (Table 2).

**Table 2:** Statistics of persistence and mechanical aperture from the scanline surveys from rock faces

Type of discontinuity	Code	Population (*n*)	Persistence (m)			Mechanical aperture (mm)		
			Range	Arithmetic mean	Standard deviation, *σ* (m)	Range	Arithmetic mean	Standard deviation, *σ* (mm)
Joints	D1, D2	157	0.07–3.90	0.66	0.08	0–2.2	0.6	0.6
Fault-related fractures	D3, D4	105	0.01–3.60	0.62	0.10	0–60.0	1.7	6.1
Bedding planes	D5, D6	114	0.60–60	3.42	0.20	0–2.5	0.7	0.6
Stylolites	D7, D8	26	0.06–6.00	0.62	0.10	0–0.9	0.3	0.3

The infilling of geological discontinuities (*n* = 401; D1–D6) has also been recorded in the scanline surveys. Such discontinuities have been divided into clay (35%), calcite-dolomite (15%), and clean (49%) discontinuities.

### Physiochemical properties of groundwater

**Fig. 5:**
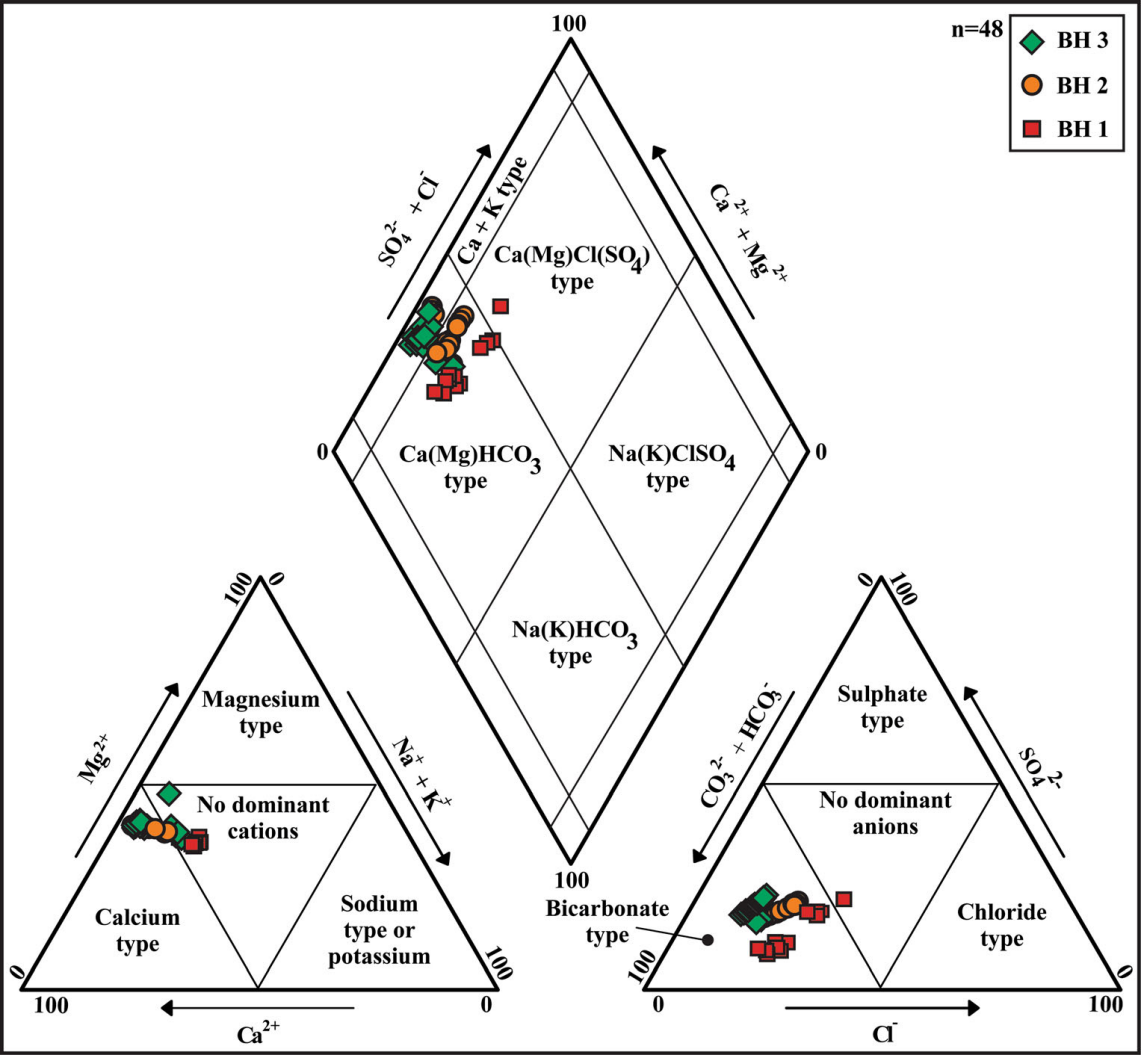
Piper diagram for water samples from BH1, BH2, and BH3

**Fig. 6:**
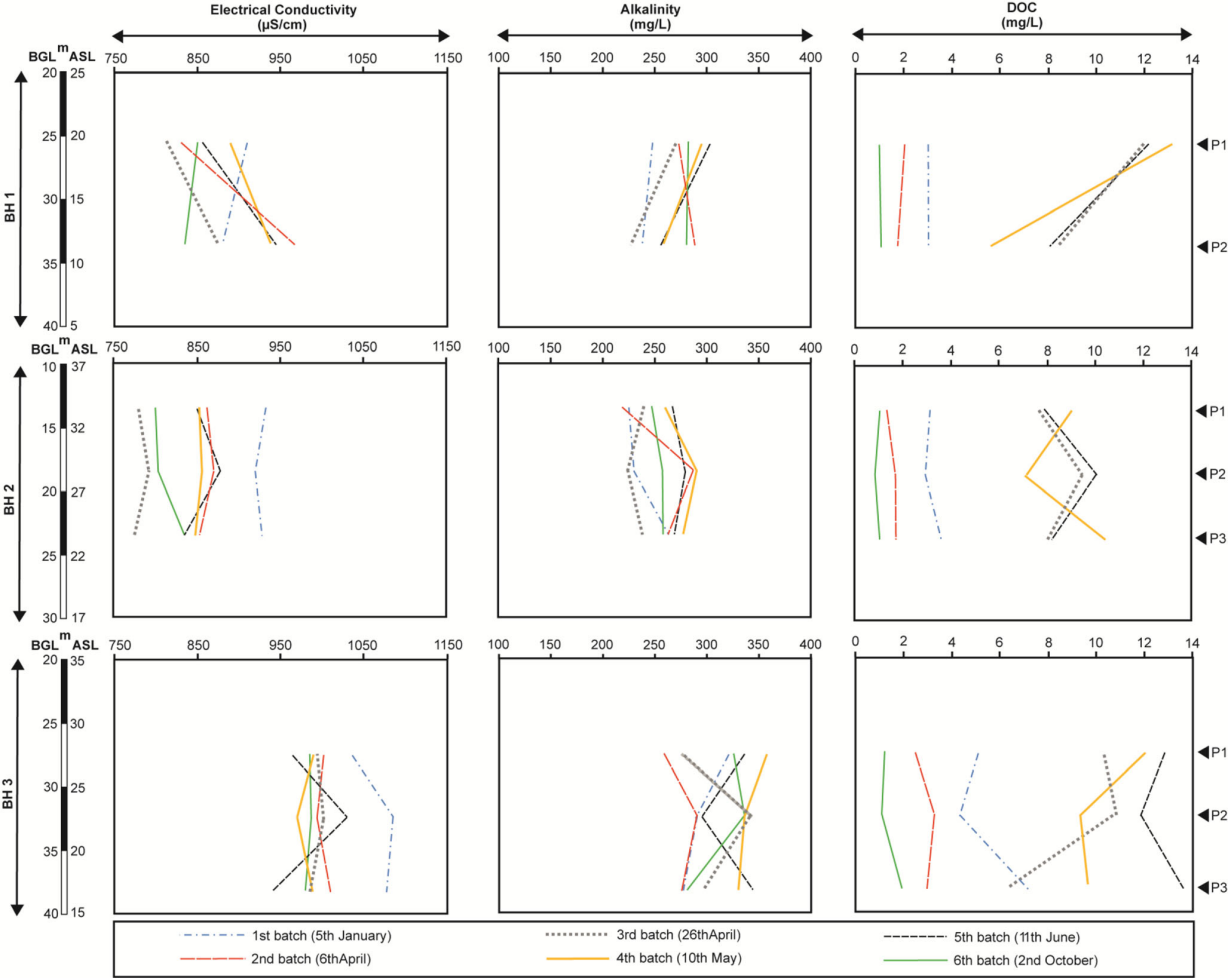
Plots of electrical conductivity, alkalinity, and DOC measured on six dates during 2018 versus depth of piezometer interval in BH1, BH2, and BH3 boreholes

Physiochemical parameters of groundwater were determined on six occasions during the hydraulic year (Jan–Oct, see hydraulic head variation vs. sampling times in Fig. S2) in BH1, BH2, and BH3 boreholes. Results from this monitoring are summarized in Figs. 5 and 6 and Table 3. The Piper diagram (Fig. 5) shows a Ca^2+^-Mg^2+^ bicarbonate-type composition. The order of abundance is Ca^2+^ > Mg^2+^ > Na^+^ > Al^3+^ > Mn^2+^ > Fe^2+^ and HCO^3−^ > SO4^2−^ > Cl^−^ > NO_3_^−^ for cations and anions, respectively. Nitrate concentrations are always above the WHO drinking water limit of 50 mg/L in BH2 (55–118 mg/L) and BH3 (60–125 mg/L); in BH1, they ranged from 40 to 65 mg/L.

**Table 3:** PHREEQC input (field measurements) and output parameters: mean, range, and standard deviation (*σ*)

Borehole	Screened interval	Input					Output			
		pH	Fluid temperature (°C)	Alkalinity (mg/L)	Ca^2+^ (mg/L)	Mg^2+^ (mg/L)	SI_Calcite_	SI_Aragonite_	SI_Dolomite_	pCO_2_
		Mean; range; *σ*	Mean; range; *σ*	Mean; range; *σ*	Mean; range; *σ*	Mean; range; *σ*	Mean; range; *σ*	Mean; range; *σ*	Mean; range; *σ*	Mean; range; *σ*
BH1	P1	7.26; 7.04–7.60; 0.2	10.3; 9.5–11.1; 0.6	289; 268–320; 16.4	94; 83–97; 4.5	43; 39–44; 1.5	0.08; − 0.19 to 0.41; 0.18	− 0.12; − 0.59 to 0.25; 0.25	− 0.02; − 0.35 to 0.60; 0.30	0.017; 0.007–0.023; 0.01
	P2	7.30; 7.20–7.53; 0.1	10.1; 9.4–11.0; 0.5	251; 217–280; 18.7	94; 83–100; 5.9	44; 38–48; 2.8	0.01; − 0.21 to 0.20; 0.15	− 0.14; − 0.63 to 0.05; 0.14	− 0.18; − 0.63 to 0.23; 0.28	0.014; 0.009–0.023; 0.01
BH2	P1	7.13; 7.01–7.38; 0.1	10.3; 9.6–11.4; 0.6	260; 241–275; 11.8	99; 90–102; 4.2	45; 41–48; 2.2	− 0.08; − 0.23 to 0.19; 0.14	− 0.24; − 0.19 to 0.21; 0.14	− 0.38; − 0.64 to 0.17; 0.28	0.017; 0.010–0.023; 0.00
	P2	7.25; 7.25–7.43; 0.1	10.2; 9.5–11.4; 0.6	256; 220–291; 15.0	99; 90–103; 4.1	45; 41–47; 2.0	− 0.16; − 1.28 to 0.27; 0.51	− 0.32; − 1.43 to 0.12; 0.51	− 0.53; − 0.26 to 0.32; 1.02	0.010; 0.001–0.018; 0.00
	P3	7.33; 7.12–7.47; 0.1	10.2; 9.5–11.2; 0.5	273; 239–304; 20.8	104; 90–130; 12.3	47; 42–56; 4.4	0.16; − 0.06 to 0.34; 0.53	0.00; − 0.22 to 0.18; 0.53	0.07; − 0.35 to 0.34; 1.03	0.012; 0.008–0.015; 0.00
BH3	P1	7.33; 7.10–7.50; 0.1	10.3; 9.5–11.4; 0.7	311; 264–349; 31.0	131; 120–137; 5.4	55; 50–58; 2.5	0.29; 0.16–0.46; 0.12	0.13; − 0.04 to 0.24: 0.12	0.33; − 0.01 to 0.67; 0.22	0.014; 0.008–0.026; 0.01
	P2	7.33; 7.15–7.56; 0.1	10.1; 9.4–11.3; 0.7	315; 273–342; 24.2	131; 124–136; 5.3	55; 52–58; 2.3	0.73; 0.14–2.27; 0.89	0.57; − 0.01 to 2.54: 0.89	1.16; 0.02–4.91; 1.70	0.013; 0.009–0.022; 0.00
	P3	7.25; 7.12–7.35; 0.1	10.0; 9.2–10.9; 0.6	308; 280–343; 22.0	118; 99–130; 3.6	52; 46–55; 1.9	0.12; − 0.03 to 0.23; 0.11	− 0.04; − 0.19 to 0.07; 0.11	0.01; − 0.27 to 0.60; 0.20	0.018; 0.013–0.023; 0.01

The mean pH of groundwater was 7.30 (range 7.01–7.60); mean fluid temperature was 10.0° (range 9.5–11.0°). Electrical conductivity ranged from 775 to 1086 μS/cm (Fig. 6). DOC ranges from 1.0 up to 13.8 mg/L (Fig. 6) and shows a wider seasonal variation than the other parameters with higher concentrations observed in the spring-summer period (late April–June).

Saturation indexes (SI) for calcite, dolomite, and aragonite and pCO_2_ computed from concentrations of Ca^2+^ and Mg^2+^, alkalinity, pH, and fluid temperature (Table 3) ranged from 0.001 up to 0.026. These values are higher than the atmospheric pCO2 of 0.00035 as is common for soil and groundwater (Talling 2006). Saturation indexes (SI) of calcite, aragonite, and dolomite lie in the range − 0.5 to 0.5 (Table 3), which indicates saturation for these three carbonate mineral species (Bicalho et al. 2012).

## Groundwater flow and transport model

**Fig. 7:**
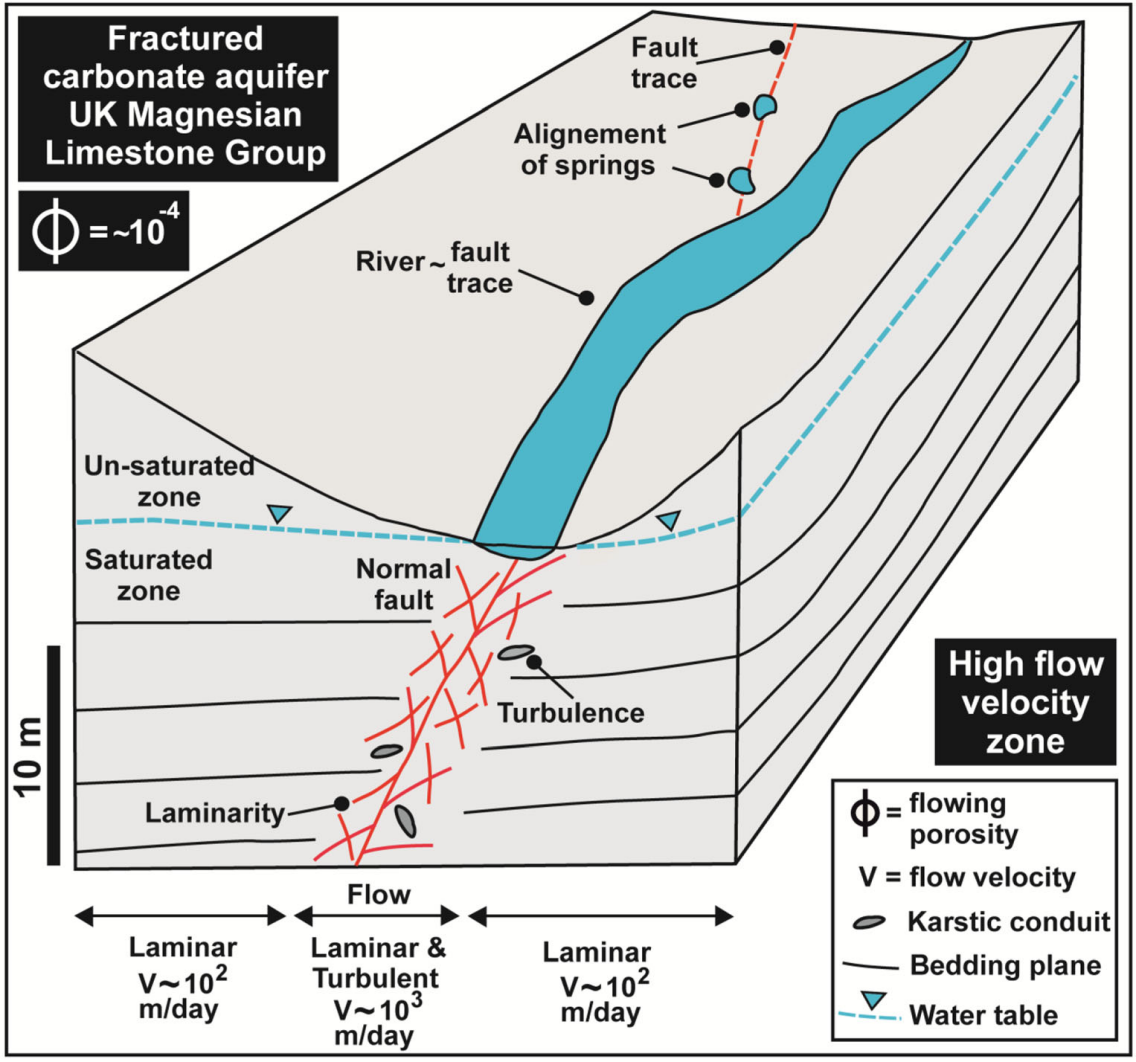
Conceptual model of groundwater flow in the fractured and faulted carbonate aquifer of the Magnesian Limestone Group

**Fig. 8:**
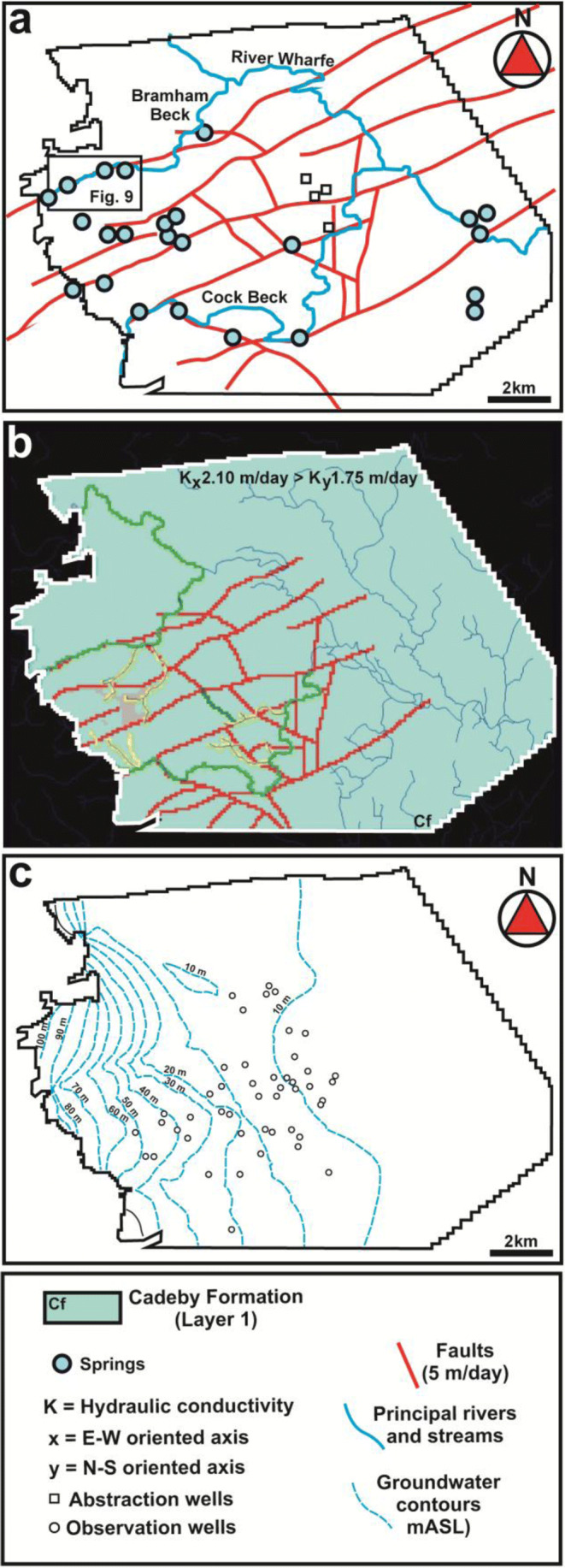
3D steady-state groundwater flow model of the Magnesian Limestone in the Leeds-York area. **a** Model area showing rivers, location of springs, abstractions wells, and mapped fault traces. **b** Cadeby Formation, layer 1 (the main flowing layer), red lines are fault cells, green are stream cells, and yellow are drain cells. **c** Calibrated model hydraulic heads (ASL) within the Cadeby Formation, lowermost layer (layer 1)

The revised conceptual model of the Cadeby Formation is shown in Fig. 7 (for the upper ~ 15 m below the water table which is significantly permeable). Extensional faults are characterized by partially closed fractures (*a* < 0.001 m) and relatively large (*a* = 0.1–0.60 m) pipes (conduits), which in some places are in alignment with springs and streams (Figs. 7 and 8a). Particle tracking analysis was realized from the new groundwater flow model.

### Pre-existing groundwater flow model (Environment Agency 2009)

The Environment Agency (2009) groundwater flow model covers an area approximately 16 km E-W by 13 km N-S; Fig. 1b shows the model boundaries, also shown in Fig. 8a–c. The grid consists of 185 rows and 205 columns; the finite-difference cell dimension is 100 × 100 m. The 3D grid is characterized by three layers which represented the Cadeby (lowest, layer 1), Edlington (layer 2), and Brotherton (uppermost, layer 3) formations of the UK Magnesian Limestone, respectively. Note that the water table decreases towards the east. Thus, some parts of the Brotherton Formation (layer 3) are unsaturated in the eastern area of the model. The Edlington Formation (evaporitic marl) is an aquitard represented with hydraulic conductivity ~ 10^3^ times lower than the carbonate units of the Cadeby and Brotherton formations. Vertical flow anisotropies (*K*_*x*_/*K*_*z*_ = 100 for layer 1, see Table 4) are set to typical values of fractured carbonate aquifers with flow dominated by subhorizontal bedding plane fractures (Boulton 1970; Odling et al. 2013). Layer thicknesses and position of faults (Fig. 8a) are based on the British Geological Survey 3D geological model (Cooper and Lawley 2007) which is built on 5 seismic lines and 300 and 55 boreholes which penetrate the Quaternary sequence and UK Magnesian Limestone, respectively (Cooper and Lawley 2007; EA 2009). Mapped fault traces are represented as very high hydraulic conductivity cells within the Cadeby Formation as shown in Table 4.

**Table 4:** Hydraulic conductivity values of the Cadeby Formation (layer 3), a unique layer involved in particle tracking analysis, including median from slug tests and pumping tests and calibrated model values for previous and new steady-state models

Model area	Slug tests, Medici et al. 2019 (*K*) (m/day)	Pumping tests, Allen et al. 1997 (K) (m/day)	Model, EA 2009 (*K*_*x*_, *K*_*y*_, *K*_*z*_) (m/day)	New model, this work (*K*_*x*_, *K*_*y*_, *K*_*z*_) (m/day)
Un-faulted	1.0	1.3	1.75, 1.75, 0.0075	1.75, 2.1, 0.021
Faulted	N/A	200	500, 500, 500	5, 5, 5

### New groundwater flow model

The new 3D steady-state groundwater flow model has been re-calibrated by auto-sensitivity analysis of hydraulic conductivity of faults and layers; i.e., the model implementation is exclusively aimed at optimizing groundwater flow with respect to specific geological structures (bedding planes, non-stratabound fractures, and faults).

In the new model (see Fig. 8a–c), the greatest input to groundwater is rainfall recharge (average 134 mm/year resulting in ~ 78% of inflow). The next greatest input (representing 13%) is flow from the underlying Carboniferous strata. Other smaller inputs (9%) result from leakage from losing reaches of surface water courses. The greatest output from groundwater (50%) is the baseflow to the River Wharfe. Baseflow to the Cock and Bramham Becks makes up another 33% of the total outflows. Abstraction in the Leeds-York area (see Fig. 8a) accounts for approximately 13% of the outflows. An additional 4% is represented by flow from the aquifer across the southern general head boundary.

Previous research at the University of Leeds farm (Figs. 1b and 2a) shows a high hydraulic conductivity (*K* = 0.83–2.85 m/day) in the first ~ 15 m immediately below the water table in the Cadeby Formation (Medici et al. 2019a), with much lower values below. As a consequence, the effective thickness of the aquifer was reduced to ~ 15 m by truncation of the bottom of the Cadeby Formation (layer 1), as indicated in the conceptual model (Fig. 7).

Vertical layer anisotropy (*K*_*x*_/*K*_*z*_ = 100 for layer 1) values were unchanged from the previous model as hydrogeophysical characterization confirmed the importance of bedding fracture flow. The model hydraulic conductivity (see Table 4) has also been modified in correspondence of mapped extensional fault traces to values of 2.5 times higher than the background in zones 100 m wide, to account for high fracturing density (Table 1). The unfeasibly high hydraulic conductivity (e.g., 500 m/d, Table 4) values used in the previous model in correspondence with fault zones are represented instead using the Conduit Flow Process Mode 1 elements (pipes). This accounts for laminar and turbulent flow of relatively large (0.1–0.6 m) conduits observed in outcrops (e.g., Fig. 3d).

The new groundwater model (Fig. 8a–c) was calibrated by automatic sensitivity analysis based on hydraulic conductivity in un-faulted areas and pipe diameter in fault zones. Hydraulic conductivity of fault zone cells was fixed to 5 m/day. Conductance of pipe elements ranges from 15 to 38 m^2^/day as a function of the hydraulic gradient (which varies from 0.005 to 0.0024 across the model area, see Fig. 8c). These values of pipe conductance come from the pipe diameter of 0.20 m, determined during calibration. This process produced similar calibration statistics of the Environment Agency (2009) model, with residual mean and absolute residual mean of − 1.43 and 3.90 m, respectively. Including a horizontal anisotropy (*K*_*x*_/*K*_*y*_ = 1.2) to represent the preferential E-W joint orientation (see Figs. 4 and S1) slightly improved model calibration (Table 4). A plot of observed vs. simulated hydraulic head for 47 observation wells is shown in Figure S2. The maximum residual of 6.5 m is < 10% of the hydraulic head range. Residual mean and absolute residual mean are − 1.33 and 3.36 m, respectively (Fig. S2).

**Fig. 9:**
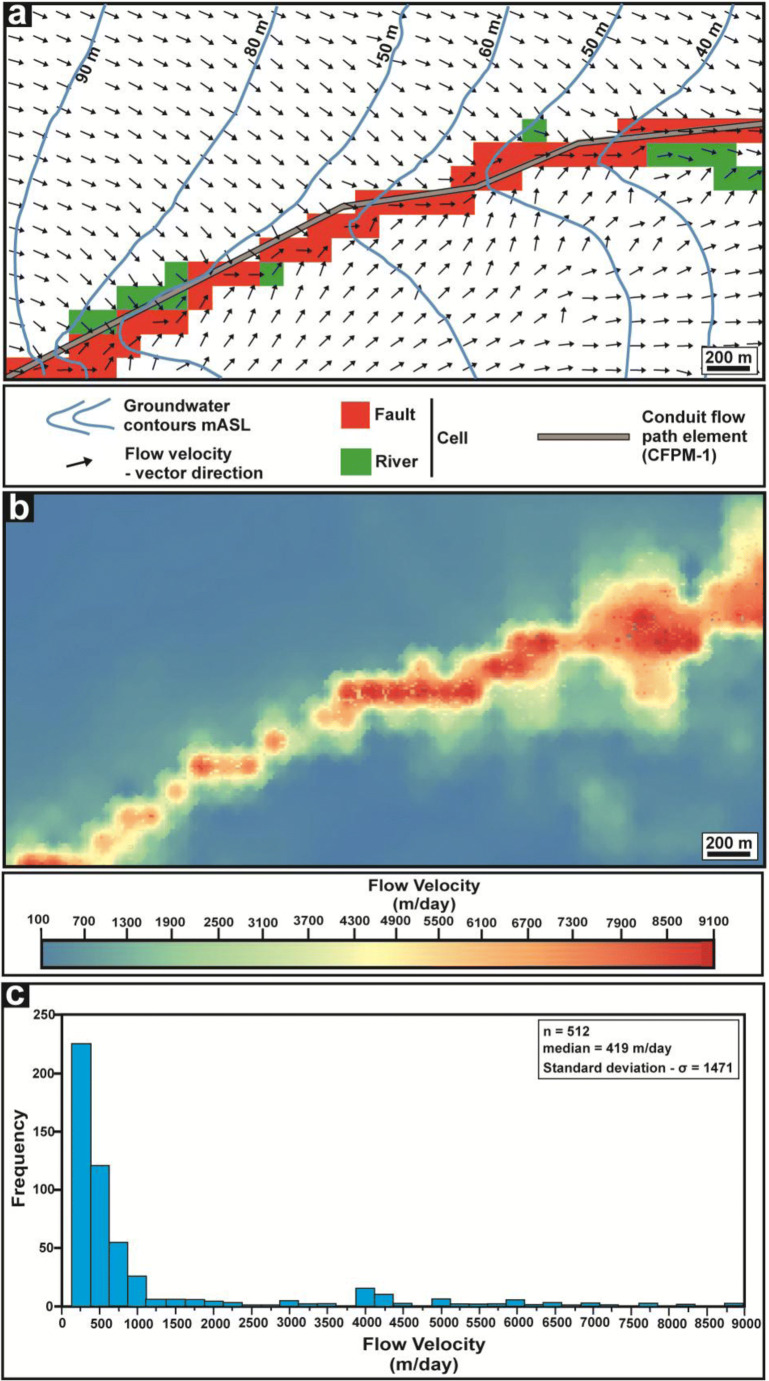
3D steady-state flow velocity field and NW model subarea (see Fig. 8a). **a** Vector flow direction and modelled fault, river, and CFPM-1 pipe element. **b** Map of flow velocity. **c** Frequency histogram of flow velocity for the subarea shown

A flow velocity field around a normal fault zone is shown in Fig. 9a–c. This is a detail of the velocity field (obtained with flowing porosity of 2.8 × 10^−4^) in the north western sector of the 3D steady-state flow (see Fig. 8a for position). Groundwater flow velocities ranged from 500 up to 9000 m/day (Fig. 9b, c) in correspondence of the fault zone. Such high values are spatially confined to the region around the conduit flow path (CFP-1) pipe element accounting for the 25% of the cells in the selected model area (Fig. 9c). The highest flow velocities (~ 1000–9000 m/day) concern cells directly in correspondence of the CFPM-1 pipes with vectors subparallel to these elements (see Fig. 9a, b). However, flow velocities rapidly decrease away from the faults, reaching values of ~ 200 m/day at ~ 300 m distance. Similar values (150–400 m/day) dominate un-faulted areas accounting for the 75% of the cells (Fig. 9c). Note that all groundwater flow velocities in this model are lower by a factor of ~ 10^2^ by alternatively using the previously published value of flowing porosity (5.0 × 10^−2^; Neymeyer et al. 2007), used for particle tracking in this carbonate aquifer of the Permian age.

### Particle tracking analysis

**Fig. 10:**
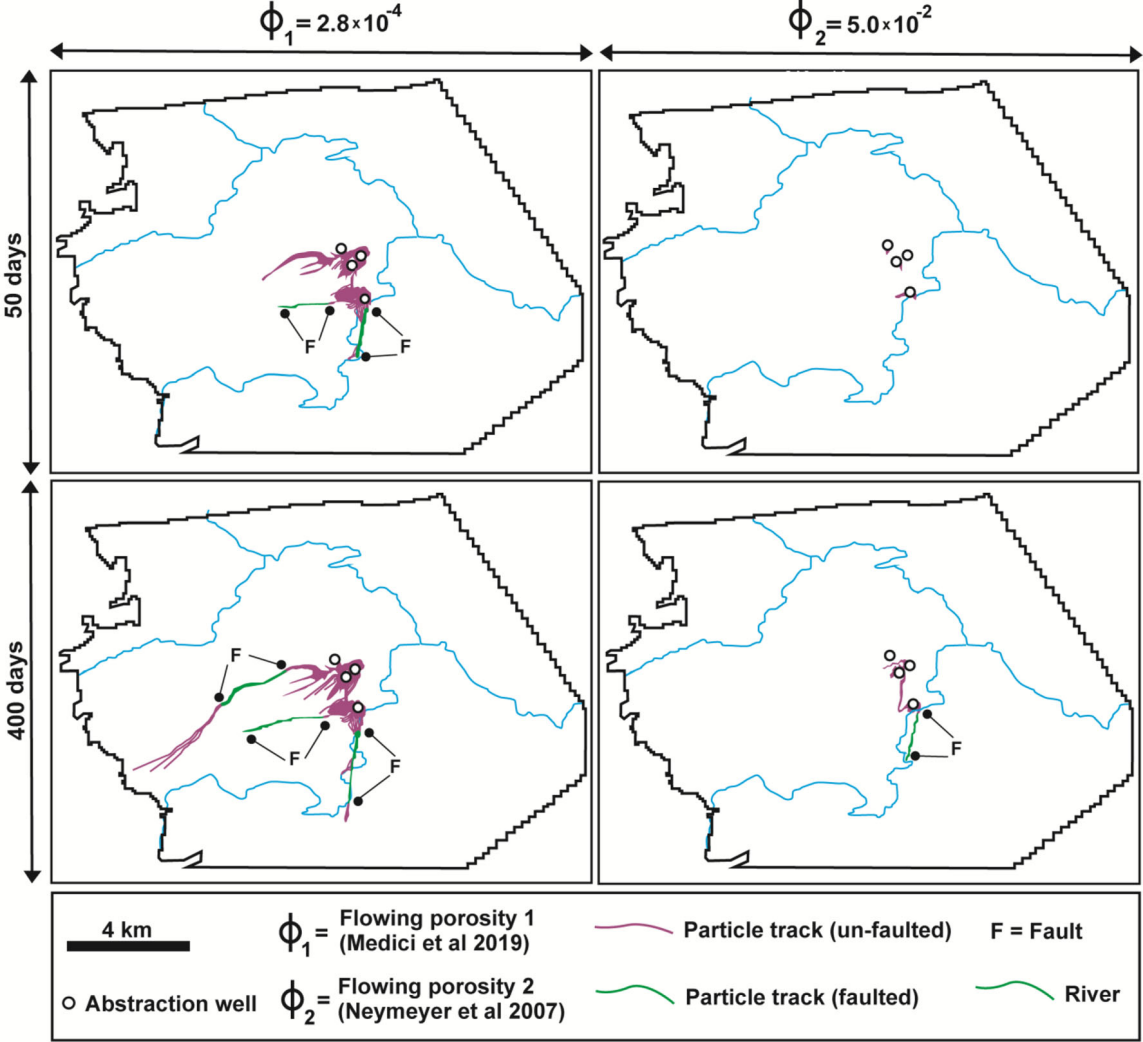
Backward particle tracking analysis from abstraction wells in the Cadeby Formation (layer 1). Left panels use the lower flowing porosity of the equivalent porous medium representing flow porosity in narrow fractures of 2.8 × 10^−4^ from hydrogeophysical characterization; right-hand panels use a more conventional higher value of 5.0 × 10^−2^

Values of effective flow porosity represent the key parameter that controls backward particle tracking as shown in Fig. 10. The upper panels show 50-day traces and the lower panels 400-day traces. The scenario shown on the left side of this figure is based on the lower value of effective flow porosity (2.8 × 10^−4^) of the non-faulted areas derived from hydraulic testing in the central part of the model area (Medici et al. 2019a). Hydrogeophysics has been specifically applied on the Cadeby Formation which is the unique layer involved in particle tracking. Hence, this scenario is more likely to be correct. In that case, karstified faults strongly influence particle tracks. Indeed, particles can reach faults and are then rapidly transported along segments of such tectonic structures (see Fig. 10, left side). Note that the low values of effective flowing porosity show evidence of hydraulic connection between abstraction wells and streams via faults (Fig. 10). Particles are transported at high velocity (~ 10^3^ m/day) until the flow vectors bring them to an area not affected by CFPM-1 pipe elements.

In contrast, using the higher effective porosity (5.0 × 10^−2^) from the Neymeyer et al. (2007) study (Fig. 10, right side of panels) does not produce traces long enough to reach the fault zones in 50 days, although some are reached in 400 days. This highlights that identification of the flowing porosity of the un-faulted areas is critical for correctly predicting aquifer vulnerability.

Notably, MODPATH was initially run using the very high values of fault zone hydraulic conductivity in the pre-existing model (*K* = 10–1000 m/day; Environment Agency 2009) rather than the CFPM-1 elements. This approach equally produces particle tracks subparallel to faults which spatially localize particles. However, the latter flow velocity field around and within fault zones is incorrect because flow velocities are too high for Darcian flow.

## Discussion

### Groundwater chemistry and karstification

Hydrochemistry data show a Ca^2+^-Mg^2+^ bicarbonate-type composition for the groundwater in the Cadeby Formation of the Magnesian Limestone Group. This is the typical composition of groundwater in dolostones and dolomitic limestone aquifers (Falcone et al. 2008; Mudarra and Andreo 2011, Xanke et al. 2015; Palmucci et al. 2016; Zhang et al. 2016). Saturation indexes computed for calcite, aragonite, and dolomite (Table 3) indicate that the groundwater is saturated with respect to these three mineral species. Despite this, the UK Magnesian Limestone shows karstic fracture aperture enhancement in the top ~ 15 m below the water table, as well as karst conduit features as a result of faults which are represented in the new groundwater flow model. Such karstic development is most likely driven by in situ bacterial consumption of DOC present in the groundwater, derived from the soil zone, as well as external input of CO_2_ via the gas phase from the soil zone. Holden et al. (2019) observed that arable practices, such as ploughing, increased decomposition rates of soil organic matter that leads to an increase in the release of carbon dioxide. Our hydrochemical analyses (see Table 3) confirm this scenario. The fact that pCO_2_ is higher in the groundwater of the Cadeby Formation with respect to the atmosphere leads to the pH of water being lower than the value of 8.3 in equilibrium with atmospheric CO_2_ (Table 3). These conditions will lead to continued dissolution of the limestone/dolomite, creating karst porosity along fractures and conduits.

Dolomitic rock observed in the field is weaker in correspondence of faults as a consequence of cataclasis and higher fracturing density (Table 1). This favours localization of streams in correspondence of normal faults as depicted and modelized in Figs. 7 and 10, respectively. As a consequence, more intense groundwater dissolution occurs due to allochthonous groundwater from streams which are characterized by much higher pCO_2_ (Banks et al. 1995; Worthington and Ford 2009; Gulley et al. 2015). Cavities up to 0.60-m diameter found at the disused quarries investigated in correspondence of faults (Figs. 1b and 3c, d) may result from such dissolution. Breccia pipes previously observed in quarries in the Leeds-York area and dolines mapped in correspondence of fault traces in the Cadeby and Brotherton formations ~ 40 km south with respect to the model area are further indicators of macro-karst development (Murphy 2000; Cooper and Lawley 2007; Farrant and Cooper 2008). Here, we have attempted to accurately model groundwater flow in a highly fractured and karstified area of carbonate aquifers affected by such extensional faults.

### Effective porosity and particle tracking analysis

This research has shown that effective flowing porosity is a key parameter for particle tracking analysis. However, a wide range of effective flowing porosity values have been reported in the literature even for the same aquifer (see Fig. 10). The low average value (2.8 × 10^−4^) that was determined by Medici et al. (2019a) for the UK Magnesian Limestone followed rigorous approaches that were recently used for fractured aquifers in Northern America (Quinn et al. 2011; Maldaner et al. 2018; Ren et al. 2018). All these studies couple acoustic televiewer logs with fluid or dilution tests to individuate flowing fractures. Then, the cubic law is applied to calculate the average hydraulic aperture using slug or pumping tests. The effective flowing porosity (2.8 × 10^−4^) value which is illustrated in the conceptual model in Fig. 7 closely matches that one found in the Palaeozoic granites of Wyoming (3.9 × 10^−4^, Ren et al. 2018) and the Permian dolostone of Ontario (2.6 × 10^−4^, Quinn et al. 2011; Maldaner et al. 2018). Note that the use of the cubic law to extrapolate hydraulic apertures (0.10–0.54 mm) in the Cadeby Formation is also broadly supported by outcrop analyses reported here; i.e., 80% of discontinuities recorded in non-faulted areas have mechanical apertures < 1 mm, closely matching hydraulic apertures.

This contrasts with the flowing porosities (~ 10^−1^–10^−3^) used for MODPATH particle tracking previously published for fracture-flow carbonate aquifers (e.g., Rayne et al. 2001; Neymeyer et al. 2007; Bredehoeft and King 2009; Yager et al. 2013; Zuffianò et al. 2016; Gárfias et al. 2018). Indeed, 5.0 × 10^−2^ has been used for the Cambrian-Ordovician limestone of Nevada (Bredehoeft and King 2009), the Permian dolostone of NE England (Neymeyer et al. 2007), the Jurassic limestone of Southern Italy (Zuffianò et al. 2016), and the Cretaceous dolostone of southern Spain (Gárfias et al. 2018). However, this relatively high value (5.0 × 10^−2^) of effective porosity appears unsupported by hydrogeophysical borehole testing. A similar effective porosity (1.0 × 10^−2^) was used for particle tracking analysis in the Palaeozoic fractured carbonates of West Virginia (Yager et al. 2013). Use of these higher values appears to lead to large underestimation of contaminant transport rates in such porous geological media. As a consequence, we believe that much more experimental efforts are needed to determine more accurate effective flow porosity values, which have been typically either subject to uncertainty or overestimated by groundwater modellers.

### Structural geology and particle tracking analysis

Backward particle tracking analysis (see Fig. 10) in the UK Magnesian Limestone of NE Yorkshire has highlighted the important role that extensional faults play as preferential pathways for contaminant transport. This arises from their much higher (~ 10^3^ m/day) groundwater flow velocity due to the higher fracturing density coupled with karstic macro-porosity structures (see Fig. 7). From a conceptual point of view, normal faults must be modelled accounting for their high hydraulic conductivity away from such conduits due to the presence of high-density less intensively karstified fractures. Indeed, a hydraulic conductivity higher than that of the un-faulted Cadeby Formation has been used in the groundwater flow model (Table 4). Discontinuity surveys performed in the vicinity of a normal fault in the UK Magnesian Limestone Group (see Fig. 3c) show that 58% of fractures are characterized by mechanical aperture < 1 mm. These values which are likely to be enhanced by unloading at quarry faces still suggest a laminar flow regime. Borehole testing reported by Medici et al. (2019a) indicates laminarity at hydraulic apertures from 0.10 up to 0.54 mm.

Despite this, a major part of fault permeability is likely to be related to a network of pipes. Cavities with rounded and angular shapes (see Fig. 3d) were recognized in outcrops in the UK Magnesian Limestone Group. Such structures typically represent fault jogs enlarged by groundwater alteration (Billi et al. 2007; Woodcock and Mort 2008). Cavities with 0.1- to 0.60-m diameter have been recognized in fault zones in this Permian aquifer. A 0.20-m diameter has been found to model flow in the CFPM-1 pipe flow framework following model calibration. Such diameters suggest turbulence based on Eqs. (1) and (2) given the hydraulic gradients present. A similar hydrostructural pattern with cavities (Fig. 3d) and springs (Figs. 7 and 8a) in correspondence of faults has been recognized in many geological realms of the word such as the Carboniferous limestones of Western Ireland (Gillespie et al. 2001; Perriquet et al. 2014), in the Triassic dolomitic limestones in Austria (Bauer et al. 2016), and in the Jurassic limestones of Central and Southern Italy (Galdenzi and Menichetti 1995; Billi et al. 2007; Petrella et al. 2007; Barberio et al. 2017). Notably, the coupling of low values of effective flowing porosity and rigorous modelling of fault permeability (see the left side of Fig. 10) show a hydraulic scenario which links abstraction wells to losing streams via faults. As a consequence, the vulnerability of heavy tectonised carbonate aquifers to contamination is very high. For example, pathogens transported by rivers are easily intercepted by abstraction wells by rapid transport through bedding plane fractures, subvertical joints, and fault-related fractures.

## Conclusion

We use the example of the Permian Magnesian Limestone aquifer in the Yorkshire area (NE England, UK) to identify the drivers of karstification in the aquifer and demonstrate the importance of both rigorous determination of the flowing fracture porosity and representation of turbulent flow in karst conduits when simulating contaminant transport in fractured limestones. In this paper, we modified a previous steady-state flow model of this carbonate aquifer using data from both hydrogeophysical borehole testing and outcrop characterization and used more rigorous approaches to model groundwater flow in and around karstic cavities. The field, experimental, and modelling results of our research can be summarized in three key points:

1. The Permian Magnesian Limestone represents an example of both fractured and karstified carbonate aquifer types. Indeed, hydrochemistry analyses indicate a Ca^2+^-Mg^2+^ bicarbonate composition for groundwater. Saturation indexes of calcite, aragonite, and dolomite indicate saturation. Relatively high levels of dissolved organic carbon (DOC) derived from soil biomass were detected in groundwater; we hypothesise that decomposition of such DOC within groundwater produces dissolved CO_2_ which is responsible for karst development. This includes minor karstic enhancement of the bedding plane and joint apertures in the upper 15 m or so of the aquifer below the water table (mechanical aperture of ~ 10^−1^–1 mm), which results in a laminar flow regime away from faults, and karstic cavities (diameter of ~ 0.20 m) developed along extensional faults, which likely results in a turbulent flow regime.
2. A flow model of the aquifer was developed to include a pipe network representing fault conduits which accounts for flow turbulence, coupled with an equivalent porous medium representing flow in highly conductive (but Darcian) flowing fractures. Particle tracking using the resulting flow field shows that flow in normal faults in the UK Magnesian Limestone aquifer will strongly influence well protection areas.
3. The most important parameter in particle tracking analysis remains the effective flow porosity for the equivalent porous medium (representing flow in narrow fractures). For the UK Magnesian Limestone aquifer, we recommend as most appropriate a relatively low value (2.8 × 10^−4^) of effective flow porosity based on borehole hydrogeophysical testing. This contrasts with previous particle tracking analyses on both the UK Magnesian Limestone aquifer or many other analogous aquifers, where values of effective porosity ~ 10^2^ times higher than that used this work most likely resulted in highly non-conservative estimates of groundwater vulnerability.

Following this research, we envisage further efforts on hydraulic characterization of faults and effective flow porosity for fractured media prior to the development of the conceptual and numerical models. This will lead to more reliable representation of groundwater flow and contaminant transport in carbonate aquifers in the subsurface.

## Electronic supplementary material

Fig. S1 Stereoplots (upper hemisphere, equal area) of discontinuities from quarries in the Leeds-York area. See Table 1 for scanline codes; Table 2 for key to discontinuity codes D1-D6. (PDF 835 kb)

Fig. S2 Calibration data; **a** observed vs. modelled heads, **b** residual heads for the Cadeby Formation (Layer 3). (PDF 1354 kb)
