# Supplementary figures and images for: Prediction of contaminant transport in fractured carbonate aquifer types: a case study of the Permian Magnesian Limestone Group (NE England, UK)
Source: Environ Sci Pollut Res Int. 2019 Jun 25;26(24):24863–84. doi: 10.1007/s11356-019-05525-z (PMC6689290; doi:10.1007/s11356-019-05525-z)

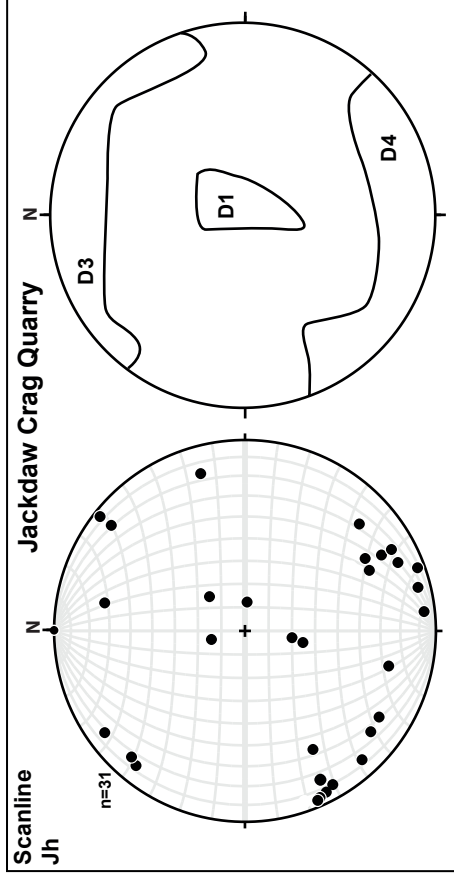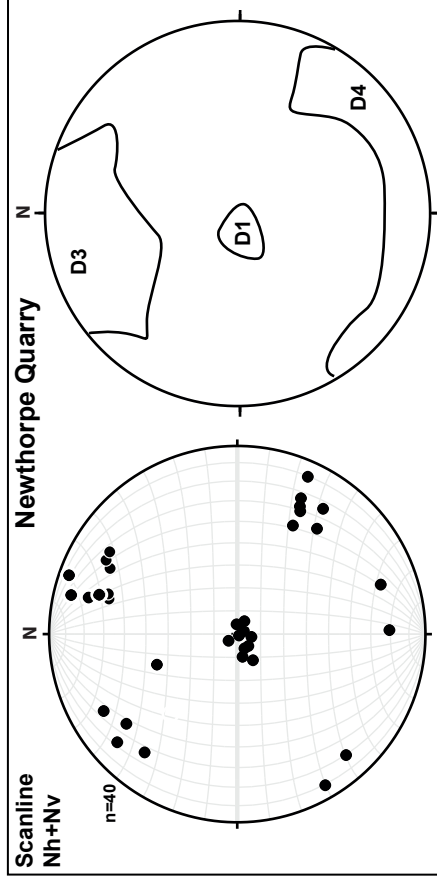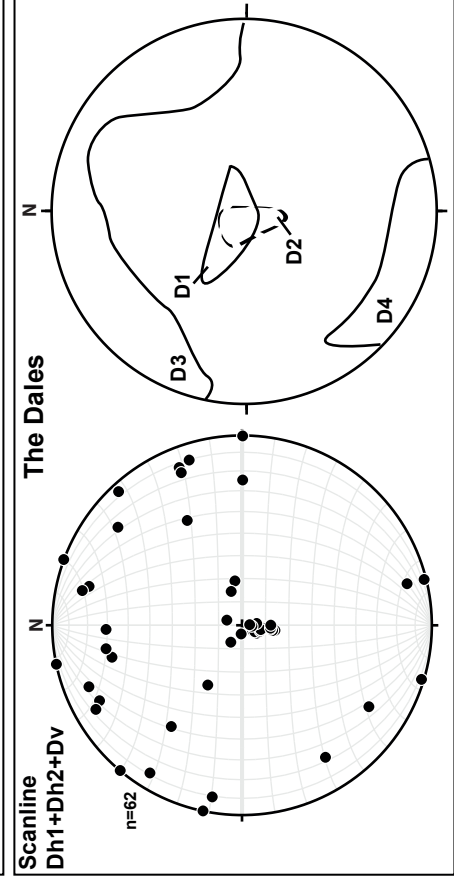

Supplement: Supplementary file 1 — Stereoplots (upper hemisphere, equal area) of discontinuities from quarries in the Leeds-York area. See Table 1 for scanline codes; Table 2 for key to discontinuity codes D1-D6. (PDF 835 kb) [file 11356_2019_5525_MOESM1_ESM.pdf]

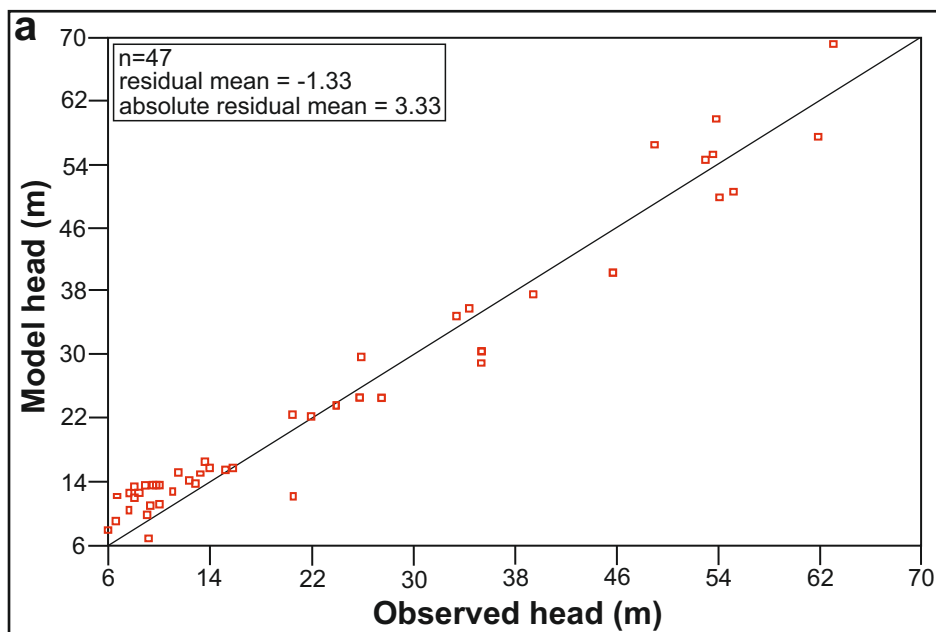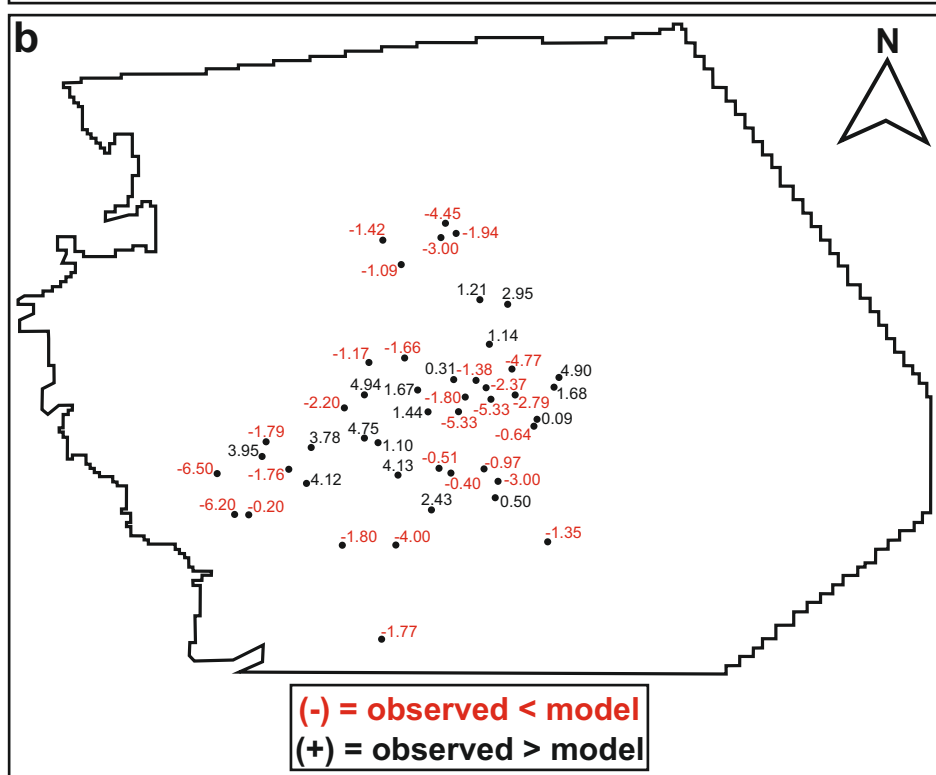

Supplement: Supplementary file 2 — Calibration data; a observed vs. modelled heads, b residual heads for the Cadeby Formation (Layer 3). (PDF 1354 kb) [file 11356_2019_5525_MOESM2_ESM.pdf]
